# A standard gamble study to determine health state utilities associated with seizures in glioma in the UK

**DOI:** 10.1186/s12955-025-02348-0

**Published:** 2025-03-11

**Authors:** Tomos Robinson, Matthew Breckons, Helen Bulbeck, Michael D. Jenkinson, Robin Grant, Luke Vale

**Affiliations:** 1 Newcastle University, Newcastle-Upon-Tyne, UK; 2 NIHR Applied Research Collaboration North East and North Cumbria, Newcastle-Upon-Tyne, UK; 3 Brainstrust-The Brain Cancer People, Cowes, UK; 4 University of Liverpool, Liverpool, UK; 5 University of Edinburgh, Edinburgh, UK; 6 Western General Hospital, Edinburgh, UK; 7 London School of Hygiene & Tropical Medicine, London, UK

## Abstract

**Background:**

Glioma are infiltrative primary brain tumours, which despite treatment, lead to a substantial reduction in life expectancy. Seizures are a common symptom of glioma, and have a serious impact on patient health related quality of life (HRQoL).

**Objective:**

The study aimed to estimate health state utility values for different types of seizures related to glioma, a serious type of brain tumour.

**Methods:**

Vignettes for the different health states were initially developed from the existing literature. The health states were then refined in collaboration with patients with previous experience of seizures and clinicians experienced in treating patients with seizures. The final vignettes represented three types of acute seizure: focal aware, focal impaired awareness and tonic clonic and several different health states which combined these acute seizures with other aspects of HRQoL. These vignettes were evaluated by a sample of the UK general public using an online survey and analysed descriptively using the mean and standard deviation.

**Results:**

302 participants, representative of the UK general population in terms of age, sex and geographical region, were included in the estimation sample. For the health states representing acute seizures, the focal aware seizure had the highest mean utility value (0.607), followed by the impaired awareness seizure (0.593) and the tonic clonic seizure (0.522). For the health states that also incorporated wider aspects of HRQoL, the health state utility values ranged from 0.504 (one focal aware seizure per year) to 0.337 (at least one focal impaired awareness seizure per week).

**Conclusions:**

Seizures may have a major impact of the HRQoL of patients with glioma. The utility values obtained in the study may be used in future economic evaluations of interventions related to glioma where seizures are either a primary clinical outcome or an adverse event.

**Supplementary Information:**

The online version contains supplementary material available at 10.1186/s12955-025-02348-0.

## Introduction

Gliomas are the most common type of primary brain tumour, with approximately 6,000 new cases diagnosed in the UK each year [1]. 20% of patients who have a suspected glioma will present with a new onset seizure prior to surgery. Of the remaining 80%, seizures will also occur, post-surgery or at some later stage prior to death in 30–50% of cases [2]. Seizures are characterised clinically as intermittent stereotyped disturbance of consciousness, behaviour, emotion, motor function or sensation. The most common types of seizure in glioma are focal with retained awareness, focal with loss of awareness, and focal with secondary generalisation (tonic clonic). Seizures may result in injuries or life-threatening complications such as status epilepticus and may also restrict a patient’s independence, for example, in the UK driving is usually prohibited for 12–24 months after a seizure, depending on the specific type and cause [3].

Seizures also impact individuals by affecting Health Related Quality of Life (HRQoL). HRQoL is an important concept in both comparative effectiveness research and economic evaluation and a key input in cost-utility analysis (CUA) where the benefits from a health care intervention are expressed in Quality Adjusted Life Years (QALYs). In health technology assessment (HTA), it is usual practice to derive utilities using for QALYs through the use of generic preference-based measures (GPBMs) [4], such as the EQ-5D [5] collected as part of, for example, randomised controlled trials (RCTs) at fixed time points through the study. However, there are several situations in which it may be inappropriate or unfeasible to collect utility data from patients. In the specific clinical context of this study, those who suffer from seizures will not be able to complete a HRQoL questionnaire such as the EQ-5D-5L during a seizure and are almost equally as unlikely to complete a questionnaire during the post-ictal period [6].

One alternative method of gathering health state utilities is with vignettes (also known in this context as “health state vignettes”, “health states”, or “health state descriptions”). A vignette is defined as a description of a health state that is valued in a preference elicitation task in order to obtain a utility estimate Matza et al. [7]. Several studies have previously used vignettes to derive utilities across a wide range of clinical conditions [8–10]. There are several circumstances in which a vignette-based method may be more appropriate than the use of a GPBM, including isolating the utility impact of specific attributes, acute and temporary health states and health states that change over time. This may be especially relevant for seizures, as these episodic symptoms that can potentially be very severe.

Preference elicitation tasks such as the Time Trade Off (TTO) and Standard Gamble (SG) are commonly used to generate utility values for vignettes [11]. A small number of studies have previously used such methods to estimate health state utility values (HSUVs) for different health states related to seizures. Using a TTO Forbes et al. [12] estimated a reduction in seizure frequency by 50% in a hypothetical health state to be 0.285 in a UK sample. Using a general population sample in Korea In a Korean sample, Kang et al. [13] used a TTO to estimate utility scores for several different health states of epilepsy with partial seizures. The utility value for a health state representing being seizure free was estimated to be 0.899, the utility value for a health state representing a reduction of seizures by over 50% was estimated to be 0.493 and the utility value of a health state representing a reduction of seizures by under 50% was estimated to be 0.303. In a Dutch sample, de Kinderen et al. [14] used the TTO to value a set of vignettes based on clinically important epilepsy attributes. Across the 11 health states valued, the utility values ranged from 0.89 (no seizures and no side-effects) to 0.22 (two severe seizures per day and severe side effects). However, none of the previous studies have specifically estimated the utility of seizures for those suffering from glioma. Furthermore, although the Forbes et al. [12] study has estimated the utility for a reduction in seizures in the UK, the estimates of this single health state were based on data from only seven individuals, as the majority of participants did not understand the task.

This SG study was embedded into the SPRING trial (Seizure Prophylaxis In Glioma) [15], a multi-centre randomised controlled trial investigating the efficacy of prophylactic levetiracetam in preventing seizures for patients with newly diagnosed cerebral glioma. The aim of this study was to estimate utility values for a range of seizure focussed vignettes. We contribute to the literature by estimating utilities for two types of seizure. First, we aimed to estimate utilities for vignettes which captured the acute symptoms of three common types of seizure, to measure the short-term utility related to acute seizures to more accurately measure HRQoL across the trial period as part of the within-trial economic analysis. Second, we aimed to estimate utility values for a set of vignettes which captured both the acute symptoms *and* wider impacts on HRQoL of seizures, for use in a long-term economic model partially based upon the results from the within trial analysis.

## Methods

### Development of the vignettes

Two distinct sets of vignettes were generated. The first set of vignettes were designed to represent the acute medical consequences of having one of the three common seizure types in glioma patients: (i) focal aware; (ii) focal impaired awareness or; (iii) tonic clonic seizure. In order capture the wider aspects of having seizures beyond these acute symptoms, the second set of vignettes were designed to represent both the acute medical consequences of having the various types of seizure, as well as the frequency with which they occur and the impact that the seizures may have on wider aspects of quality of life, such as worry, frustration, concentration, lack of control and social isolation. As there is marked heterogeneity in the frequency and severity of seizures that people with glioma related epilepsy may suffer, a range of health states were generated to broadly cover the range of experiences that people may have.

**Fig. 1:**
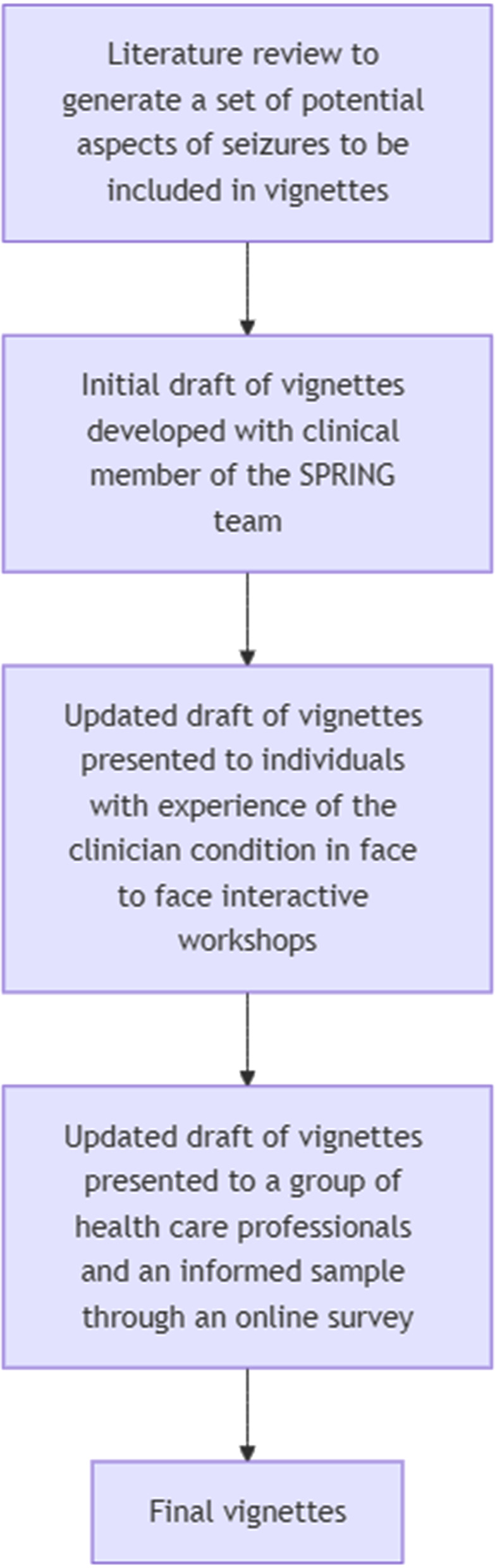
Process of generating vignettes

As shown in Fig. 1, an iterative process was used to generate the vignettes. Initially, the literature surrounding seizures was reviewed to generate a set of potential aspects of seizures that could be included. An initial draft set of vignettes was then developed with a clinician member of the SPRING trial team (RG). This initial set of vignettes was then presented to a group of individuals with experience in this condition in two interactive workshops facilitated by the brain tumour charity *brainstrust* in January 2020, which included a video recorded by the project team depicting how the vignettes would be valued as part of a SG exercise. Based on the feedback from this workshop, several minor changes were made to the wording of the vignettes, and the number of vignettes to be presented were reduced to ten to ease participant burden. As noted by Matza et al. [7] the number of vignettes developed should be partially based on the feasibility of the valuation task.

Finally, the vignettes were presented to a group of health care professionals and an ‘Informed Sample’ through an online survey coded in the software package *Qualtrics* in September 2022. The 18 health care professionals (16 Doctors and two Nurses) were gathered using the contacts of a clinician member of the SPRING trial team (RG). The six members of the informed sample (three individuals currently suffering from seizures, two individuals who previously suffered from seizures and one individual whose close member currently suffers from seizures) were once more gathered through the brain tumour charity *brainstrust*, but did not take part in the previous interactive workshops. Based upon feedback several minor changes were made to the wording of the health states.

### Standard gamble

In order to value the vignettes, the SG suite of methods were used. The SG is a well-established way of measuring the utility of specified health states [16] and is generally considered to have the strongest theoretical background of the choice-based valuation methods commonly used to weight different health states, as it is based upon the expected utility theory of rational decision-making under uncertainty or risk [17]. In this study we chose the SG suite of methods over the TTO as it was felt that dealing with risk rather than trading days of life was easier to consider in this specific clinical context given the short, intermittent nature of seizures.

#### Conventional SG

In a conventional SG exercise, an individual is asked to choose between two hypothetical alternatives. The first alternative is a certain health state (i) for a given period of time. The second alternative is a ‘gamble’, where the individual has a chance (p) of a restoration to perfect health, and a complementary probability (1-p) that they will die immediately. The respondent is asked the minimum value of p they would accept to be indifferent between the two alternatives. The utility of health state in question is therefore given by *p*:

1 $${H_i}={\text{ }}p$$

#### Chained SG

For the acute health states presented to the respondents (i.e. the health states that present the clinical symptoms of the seizure and do not include the frequency of the seizures or any broader effects on HRQoL), the ‘chained’ SG was used. A variant of the conventional SG, the chained SG has been designed to take account of the fact that the conventional SG may be less appropriate when measuring temporary health states, chronic health states or health states which are associated with a high level of utility [18]. As argued by Jansen et al. [19], in some cases using the conventional SG will not constitute a realistic choice, resulting in biased estimates of the utility of temporary health states. In the context of this study, using the standard SG for a temporary health would result in a respondent trading off health states which last one day with immediate death, an extremely cognitively difficult task which will likely result in biased estimates of utility. In a chained SG, the health state being evaluated is not weighed against the extreme values of ‘perfect health’ and ‘death’ but is instead weighed indirectly through the use of an intermediate ‘anchor’ health state. As noted by Torrance [18], the anchor state should be worse than the worst health state valued in the SG exercise, but not worse than death. In this study, we used the EQ-5D-5L state 45433 as the anchor state, as this state was judged to be better than death (valued at 0.100 in the EQ-5D-3L value set currently recommended by the National Institute for Health and Care Research (NICE) for EQ-5D-5L values [20]), but worse than the worst state valued in the SG exercise. Although not as widely used as the conventional standard gamble, the chained variant of the SG has been used in several previous studies, including some studies specifically related to cancer [21–22].

The chained SG is a two-step procedure. In the first step, the respondent is asked to choose between two alternatives. The first alternative is the ‘anchor state’. The second alternative is the same as the conventional SG, where the individual has a chance (p) of a restoration to perfect health, and a complementary probability (1-p) that they will die immediately.

In the second step, the respondent is once more asked to choose between two alternatives. The first alternative is a certain health state (i). The second alternative is a gamble, where the individual has a chance (p) of a restoration to perfect health, and a complementary probability (1-p) that they will be in the anchor state valued previously. Once more, the respondent is asked the minimum value of p they would accept to be indifferent between the two alternatives.

The utility of the health state in question (*H*_*Q*_) using the chained SG is therefore given by:

2 $${H_Q}={\text{ }}p{\text{ }}+{\text{ }}(1--p){\text{ }}{H_A}$$

*H*_*A*_ represents the value of *p* calculated for the anchor health state calculated in the first part of the chained SG. *p* represents the probability for which the participant is indifferent between the alternatives in the second part of the chained SG.

#### Choice list

In this study, both the conventional and chained SG tasks were presented using a choice list format. Although this method has been used in the context of a SG [23], it has more commonly been used in studies relating to health outcomes [24, 25] and in the experimental economics literature where it is known as the Multiple Price List (MPL) [26–28]. In the context of a SG, the choice list consists of an array of ordered SG choices presented simultaneously in a single table. For each row, the respondent is asked to indicate which alternative they prefer, or whether they are indifferent between the two alternatives presented. This contrasts to the traditional SG exercise, where each individual choice is presented singularly in an iterative ‘ping pong’ format [29]. One of the main advantages of using a choice list to present the SGs in this context is that it reduces the burdensome nature of completing the SG in the usual manner in an online setting. Another advantage of the choice list format in the context of an online SG is that the choice list can be easily coded so that it prohibits violations of stochastic dominance and multiple switching within each choice list, which would result in inconsistent preferences.

For the conventional SG, the duration of Alternative A (the certain health state) was 10 years followed by death, with Alternative B being an X% chance of perfect health for 10 years followed by death and a (1-X)% chance of a painless death within one week. For the chained SG, the duration of Alternative A (the certain health state) was one day, with Alternative B being an X% chance of perfect health for one day and a (1-X)% probability of the anchor health state for one day. There were eleven rows in each choice list, with the chance of death in Alternative B ranging from 100 to 0% in 10% intervals. An example of how a choice list is displayed is shown in Appendix 1. The various health states included in both the conventional and chained SG exercises are shown in Appendix 2–3.

#### Study participants

We aimed to collect data from two distinct samples of individuals: (1) a sample of individual representative of the general population of the United Kingdom (UK) (target sample size 300) and; (2) an ‘informed sample’ with previous experience of seizures (target sample size 100). After reading a detailed participant information sheet and consenting to taking part in the study, the study participants completed screening questions related to their age, gender and geographical region. The respondents then read some further details regarding the study contents before moving on to the main part of the survey.

#### Survey design

In Section A, the respondents completed the EQ-5D-5L health questionnaire (including the EQ-VAS). The purpose of this was to introduce the concept of the valuing health states and also introduce them to the EQ-5D-5L classification system, which was used later in the survey. In Section B, the respondents were first shown a detailed description of the conventional SG (presented in a choice list) and how you might complete it, using an example health state from the EQ-5D-5L classification system (health state 23343). The respondents then completed practice conventional SG task using another example health state from the EQ-5D-5L classification (health state 12343). The respondent then completed seven conventional SG tasks using the choice list, with the order randomised to avoid any order response bias.

In Section C, the respondent first completed a conventional SG for the EQ-5D-5L health state 45433. As discussed previously, this anchor state was designed to be worse than the worst health state valued as part of the chained SG exercise, but not worse than death. The responses to this SG exercise were used as the anchor state in the chained SG. The respondent then completed the three chained SG tasks, once more using the choice list. For both the conventional and chained SG tasks, the survey company ensured that the choice lists prohibited multiple switching and violations of dominance within each choice list. In Section D the respondents answered two questions about how much they understood the SG exercises and to what extent they found them difficult. In Section E, the respondents answered a set of sociodemographic questions.

A 10% pilot sample of the general population sample (*n* = 30) was collected to ensure that the respondents responded in a logical way to the online survey and were able to differentiate between the different vignettes in the SG tasks.

#### Empirical analysis

The responses to the conventional and chained SG tasks were converted to utility scores for each individual using the formulae presented in Eq. 1 and Eq. 2 respectively, and then presented as mean values together with their associated standard deviations. Given that at the time of writing there is no UK value set for the EQ-5D-5L value set currently recommended by NICE [30], the responses to the EQ-5D-5L were converted into utility scores by mapping onto the Dolan (1997) EQ-5D-3L value set [20] using the mapping function developed by the Decision Support Unit [31], using the ‘EEPRU dataset’ Hernández Alava et al. [32]. The demographic information and the responses to the post SG questions regarding the difficulty of the task were analysed descriptively. The data were analysed using Stata version 18.1 [33].

## Results

In total, 302 members of the general population sample completed the online survey in September 2023. As no problems were found with the way the pilot sample (*n* = 30) responded to the SG tasks or the survey as a whole, no changes were made to the online survey and the 30 pilot responses were included in the full general population sample. Only eight members of the ‘informed sample’ completed the survey, and therefore the results from these respondents are not presented due to the limited sample size. The descriptive statistics of the general population sample are shown in Table 1. As shown, the sample was generally representative of the adult population of the UK. Table 2 displays a summary of the results from the EQ-5D-5L and EQ-VAS. The mean utility score (0.791) was slightly lower than a recent estimate of mean EQ-5D-5L utility published by Schneider et al. [34] (0.833) and the EQ-5D-3L population norms published by Kind et al. [35] (0.856).

**Table 1:** Descriptive statistics of Estimation sample (*n* = 302)

Variable	*N*	%
**Gender**		
Male	147	49%
Female	155	51%
**Age**		
18–24	36	12%
25–34	52	17%
35–44	54	18%
45–54	50	17%
55–64	47	16%
65+	63	21%
**Region**		
East Midlands	22	7%
East of England	25	8%
London	40	13%
North East	12	4%
North West	34	11%
South East	43	14%
South West	27	9%
West Midlands	27	9%
Yorkshire and the Humber	25	8%
Wales	14	5%
Scotland	25	8%
Northern Ireland	8	3%
**Ethnicity**		
White	254	84%
Mixed	10	4%
Asian	27	8%
Black	8	4%
Other/Prefer not to Say	3	1%
**Marital Status**		
Single	105	35%
Married/Cohabiting	166	55%
Separated/Divorced	20	7%
Widowed	10	3%
Prefer Not To Say	1	0%
**Education**		
None	1	0%
Secondary School	63	21%
College/Sixth Form	91	30%
University	136	45%
Other	10	3%
Prefer not to Say	1	0%
**Household Income**		
<£10k	38	5%
£10-20k	91	13%
£20-30k	133	19%
£30-40k	115	16%
£40-50k	72	10%
£50-60k	50	7%
£60-70k	62	9%
£70-80k	23	3%
£80-90k	24	3%
£90-100k	19	3%
£100-150k	33	5%
>£150k	9	1%
Prefer not to Say	42	6%
**Longstanding Illness**		
Yes	77	26%
No	218	72%
Prefer Not To Say	7	2%
**Variable**	**Mean**	**Standard Deviation**
**EQ-5D-5L Utility Score**	0.791	0.219
**EQ-VAS Score**	75	19

**Table 2:** Mean utility values for the acute seizure health States

Seizure Type	Mean (SD)
Focal Aware Seizure	0.607 (0.372)
Focal Impaired Awareness Seizure	0.593 (0.376)
Tonic Clonic Seizure	0.522 (0.383)

*N* = 302. Standard Deviations in parentheses.

The responses to the SG questions for each of the health state were converted into utilities using the formulae shown in Eq. 1 and Eq. 2 and are summarised in Tables 3 and 4 respectively. The mean utility value (SD) for the anchor state (EQ-5D-5L health state 45433) was 0.318 (0.328). As shown in Table 3, the utility values for the acute health states calculated using the chained version of the SG follow the pattern one would expect *a prioiri*, with the health state representing an acute focal aware seizure (the least severe) having the highest mean utility value (0.607), followed by the impaired awareness seizure health state (0.593) and the (most severe) tonic clonic seizure health state (0.522).

**Table 3:** Mean utility values for the non-acute seizure health States

	Frequency		
Seizure Type	Once Per Year	Two Seizures Per Month	At Least One Seizure per Week
Focal Aware Seizure	Health State B1 = 0.504 (0.361)	Health State B4 = 0.412 (0.346)	Health State B6 = 0.368 (0.346)
Focal Impaired Awareness Seizure	Health State B2 = 0.464 (0.360)	Health State B5 = 0.389 (0.341)	Health State B7 = 0.337 (0.331)
Tonic Clonic Seizure	Health State B3 = 0.437 (0.355)	X	X

*N* = 302. Standard Deviations in parentheses.

**Table 4:** Understanding and difficulty of tasks

Variable	*N*	%
**Understanding of Task**		
Fully Understood	195	65%
Partially Understood	97	32%
Did Not Understand	10	3%
**Difficulty of Task**		
Difficult	28	9%
A Little Difficult	141	47%
Neither Difficult Nor Easy	75	25%
Easy	43	14%
Very Easy	15	5%

*N* = 302. Standard Deviations in parentheses

As shown in Table 4, the utility values for the health states incorporating wider aspects of HRQoL calculated using the standard version of the SG also follow the pattern one would expect a priori, with the utility value decreasing as the seizures become more severe and more frequent. For example, the mean utility value of Health State B1 (one focal aware seizure per year) had a mean utility value of 0.504, while the mean utility value of Health State B2 (one focal awareness impaired seizure per year) had a mean utility value of 0.464 and Health State B4 (two focal aware seizures per month) had a mean utility value of 0.412. Health State B7 (at least one focal awareness impaired seizure per week) had the lowest mean utility value of 0.337.

Table 4 shows the responses to the post interview questions related to the understanding and difficulty of the SG tasks. As shown, 97% of respondents reported either “fully” or “partially” understanding the SG tasks, with only 3% of respondents reporting that they did not understand the task. In terms of difficulty, 56% of the respondents reported the tasks being either “difficult” or “a little difficult”, with 25% of respondents reporting the tasks as being “neither difficult or easy” and 19% of respondents reporting the tasks as being “easy or very easy”.

## Discussion

Seizures can severely impact various aspects of people’s lives, however empirical data on the HRQoL associated with different types of seizure are limited. The primary aim of this study was to calculate HSUVs for various types of seizure, both acute and combined with other aspects of HRQoL for use in future economic analyses where seizures are either a primary outcome measure (such as in the SPRING trial) or as an adverse event.

For the vignettes representing three types of acute seizure, the focal aware seizure had the highest mean utility value (0.607), followed by the impaired awareness seizure (0.593) and the tonic clonic seizure (0.522). For the vignettes that also incorporated wider aspects of HRQoL, the utility values ranged from 0.504 (one focal aware seizure per year) to 0.337 (at least one focal impaired awareness seizure per week). This relatively large decrement between health states are typical of studies in this area [12–14]. Furthermore, these differences are to be expected, given the significant difference between a rare, unexpected episode without loss of awareness (one focal aware seizure per year) and random weekly events which you may not be able to remember and also carry a risk of serious injury (at least one focal impaired awareness seizure per week). These utility estimates have the potential to be used in several different aspects of economic evaluation. In a within-trial setting, these utilities may be combined with information from the trial. In an economic modelling setting, these utility values could be used to estimate disutility values for seizures when included as an adverse event.

The relatively low utilities for the more severely impaired health states (both acute and those states which incorporate wider aspects of HRQoL) highlight the substantial impact of seizures on HRQoL. In order to further contextualise the condition-specific utilities generated from this study, it is useful to compare the utility values with equivalent utility values from a GPBM [36], despite the difficulties in this approach due to differences in measurement properties across the instruments. In this case, the utilities from the most and least severe non-acute health states were compared with those from the UK EQ-5D-3L value set [20] currently recommended by NICE. The mean value of Health State B1 (One Focal Aware Seizure Per Year) was 0.504, equivalent to the EQ-5D-3L health state 42233, implying that in this health state patients experience a level of utility equivalent to ‘moderate’ in three of the five EQ-5D-3L dimensions. In comparison, the mean value of Health State B7 (At Least One Seizure Per Week) was 0.337, equivalent to the EQ-5D-3L health state 14324, implying that in this health patients experience a level of utility equivalent to ‘severe’ problems in two of the five EQ-5D-3L states.

It is also useful to attempt to compare the results from this study to those from the previous literature. The results from the Forbes et al. [12] and Kang et al. [13] studies are not comparable with this study as the health states in generated in these studies relate to specific percentage reductions in seizure frequency and did not include seizure severity. Using the results from de Kinderen et al. [14], a health state with one ‘Type 4’ seizure a month (with side effects) is estimated to have a HSUV of 0.543. The utility value of the nearest health state to this included in this study (Health State B5) is 0.389. Furthermore, a health state with one ‘Type 3’ a week (with side effects) is estimated to have a HSUV of 0.523 using the results from de Kinderen et al. [14]. The utility value of the nearest health state to this included in this study (Health State B6) is 0.368.

Whilst it is useful to compare the results from this study to the Kinderen et al. [14] study, there are several important differences between the studies that be noted. Firstly, the Kinderen et al. [14] study used TTO rather than SG, and it has previously been found that the utilities generated using the SG method are consistently higher than those using TTO methods [37]. Secondly, as the Kinderen et al. [14] study was in a Dutch population rather than a UK population and was on average younger, more educated and had a higher and proportion of women, there may be cultural or sampling differences they may have impacted the results. Finally, a further likely reason for the difference between the respective HSUVs in this study and de Kinderen et al. [14] study is the in the wording of the vignettes themselves. There are differences in the descriptions of the seizures and the frequency of the seizures, but most importantly this study has more detailed descriptions of the impact of seizures on wider aspects of quality of life, whereas de Kinderen et al. [14] groups the side-affects together as “No to mild”, “Moderate” and “Severe”.

There are several strengths to this study. Firstly, the vignettes valued in the study were developed in collaboration with clinician experts and patients with first-hand experience of seizures to ensure they were fit for purpose. Secondly, the sample size for the general population sample was representative of the UK population and also relatively large for studies of this nature (*n* = 302), minimising the level of uncertainty regarding the utility estimates for the different vignettes. Thirdly, the use of the chained SG for the acute health states ensured that no unrealistic comparisons were made between temporary health states and death, which may have resulted in biased utility estimates for these vignettes. Fourthly, the study also provides further evidence that the choice list methodology may be an effective method to pragmatically operationalise the gathering of SG data in an online setting. Finally, the majority of the participants felt that they ‘fully understood’ the SG tasks (65%), with only 3% feeling that they did not understand. Although 56% of the respondents agreed that the SG tasks were either ‘difficult’ or ‘a little difficult’, it should be noted that the SG is a cognitively complex task which requires the careful consideration of both health status and probability, and therefore a certain level of difficulty is to be expected.

The study has some limitations. Firstly, the original intention of the study was to gather responses from both a general population and an ‘informed sample’ of participants with either first- or second-hand experience of seizures. However, we were unexpectedly unable to recruit a sufficient number of participants for the ‘informed sample’ and we were therefore unable to explore any potential differences between them. Secondly, given the applied nature of the study, we did not seek to answer the question regarding if the chained SG method is more responsive than the conventional SG in the context of temporary health states. Further research in this area is required to determine the potential difference between these two SG variants in this context. Thirdly, the presentation of the SG in the form of a choice list means that the responses could be susceptible to framing effects, with respondents drawn to the middle of the list. Fourthly, the SG methodology in general has several limitations, including the cognitive demand of considering complex probabilities and the fact that respondents may be unwilling to accept gambles with small probabilities of death due to risk aversion. It is worth noting that alternative methods such as the TTO also have a variety of limitations. Finally, as with all survey using online data collection, there is no guarantee that all of the respondents completed the survey themselves, and there is also the potential for various types of bias, including sample-selection bias and nonresponse bias.

## Conclusion

Seizures can have a major impact on the HRQoL of patients with glioma. This study used the SG suite of methods to estimate utilities for a series of vignettes related to different types of seizure for patients with glioma in the UK. The utilities followed the pattern one would expect a priori, with higher HSUVs for health states with less severe symptoms. The utilities estimated in this study would be useful in economic analyses assessing the value of treatments for seizures to inform decisions regarding healthcare resource allocation.

## Electronic supplementary material

Below is the link to the electronic supplementary material.

Supplementary Material 1

Supplementary Material 2

Supplementary Material 3

## Data Availability

No datasets were generated or analysed during the current study.
