# Supplementary material for: A standard gamble study to determine health state utilities associated with seizures in glioma in the UK
Source: Health Qual Life Outcomes. 2025 Mar 11;23:22. doi: 10.1186/s12955-025-02348-0 (PMC11900579; doi:10.1186/s12955-025-02348-0)
Supplement: Supplementary file 1 — Supplementary Material 1 [file 12955_2025_2348_MOESM1_ESM.docx]

| **Appendix 1 – Example of Standard Gamble Presentation Format Using Choice List** | | | | |
| --- | --- | --- | --- | --- |
| Alternative A | A | Can’t Choose | B | Alternative B |
| 100% Chance of Perfect Health for 10 Years  0% Chance of Immediate, Painless Death | X |  |  | Health State X for 10 Years  Followed by Death |
| 90% Chance of Perfect Health for 10 Years  10% Chance of Immediate, Painless Death | X |  |  | Health State X for 10 Years  Followed by Death |
| 80% Chance of Perfect Health for 10 Years  20% Chance of Immediate, Painless Death | X |  |  | Health State X for 10 Years  Followed by Death |
| 70% Chance of Perfect Health for 10 Years  30% Chance of Immediate, Painless Death | X |  |  | Health State X for 10 Years  Followed by Death |
| 60% Chance of Perfect Health for 10 Years  40% Chance of Immediate, Painless Death |  | X |  | Health State X for 10 Years  Followed by Death |
| 50% Chance of Perfect Health for 10 Years  50% Chance of Immediate, Painless Death |  | X |  | Health State X for 10 Years  Followed by Death |
| 40% Chance of Perfect Health for 10 Years  60% Chance of Immediate, Painless Death |  |  | X | Health State X for 10 Years  Followed by Death |
| 30% Chance of Perfect Health for 10 Years  70% Chance of Immediate, Painless Death |  |  | X | Health State X for 10 Years  Followed by Death |
| 20% Chance of Perfect Health for 10 Years  80% Chance of Immediate, Painless Death |  |  | X | Health State X for 10 Years  Followed by Death |
| 10% Chance of Perfect Health for 10 Years  90% Chance of Immediate, Painless Death |  |  | X | Health State X for 10 Years  Followed by Death |
| 0% Chance of Perfect Health for 10 Years  100% Chance of Immediate, Painless Death |  |  | X | Health State X for 10 Years  Followed by Death |
