# Supplementary material for: A standard gamble study to determine health state utilities associated with seizures in glioma in the UK
Source: Health Qual Life Outcomes. 2025 Mar 11;23:22. doi: 10.1186/s12955-025-02348-0 (PMC11900579; doi:10.1186/s12955-025-02348-0)
Supplement: Supplementary file 2 — Supplementary Material 2 [file 12955_2025_2348_MOESM2_ESM.docx]

**Appendix 2 – Health States Valued Using Conventional Standard Gamble**

**Health State A (Practice Task)**

You have no problems walking about

You have slight problems washing or dressing yourself

You have moderate problems doing your usual activities

You have severe pain or discomfort

You are moderately anxious or depressed

**Health State B1**

You have one seizure per year which lasts one minute.

When a seizure happens, a twitching begins in one hand, and then spreads up the same side of your body.

During the seizure, you stop speaking and your head turns to one side.

After the seizure you feel tired but can remember exactly what happened during the seizure.

Because you do not know when a seizure might take place, you sometimes feel worried and frustrated.

You sometimes feel socially isolated and distant from your friends and cannot take part in some of your normal activities.

You also have some problems concentrating, and do not feel fully in control of your life.

**Health State B2**

You have one seizure a year which lasts three minutes.

When a seizure happens, you first experience a feeling of déjà vu and a sudden change in your mood.

Following this, you start to chew, mumble and smack your lips together, and you are unaware of the surrounding environment.

After a seizure, you feel tired and confused, and cannot remember exactly what happened during the seizure.

Because you do not know when a seizure might take place, you sometimes feel worried and frustrated.

You sometimes feel socially isolated and distant from your friends and cannot take part in some of your normal activities.

You also have some problems concentrating, and do not feel fully in control of your life.

**Health State B3**

You have one seizure a year which lasts five minutes.

When the seizure happens, you first experience a feeling of déjà vu and a sudden change in your mood.

Following this, your muscles become tense, you lose consciousness and fall to the floor, causing bruising. Your limbs then bend and jerk quickly and repeatedly and you bite your tongue. During the seizure, you wet yourself.

After the seizure, you feel drowsy and confused, have a headache and are not able to remember what happened during the seizure.

Because you do not know when a seizure might take place, you sometimes feel worried and frustrated.

You sometimes feel socially isolated and distant from your friends and cannot take part in some of your normal activities.

You also have some problems concentrating, and do not feel fully in control of your life.

**Health State B5**

You have two seizures a month, each of which lasts three minutes.

When a seizure happens, you first experience a feeling of déjà vu and a sudden change in your mood.

Following this, you start to chew, mumble and smack your lips and you are unaware of the surrounding environment.

After the seizure, you feel tired and confused, and cannot remember exactly what happened during the seizure.

Because you do not know when a seizure might take place, you regularly feel worried and frustrated.

You regularly feel socially isolated and distant from your friends and cannot take part in a lot of your normal activities.

You also regularly have problems concentrating, and do not feel fully in control of your life.

**Health State B4**

You have two seizures per month, each of which lasts one minute.

When a seizure happens, a twitching begins in one hand, and then spreads up the same side of your body.

During the seizure, you stop speaking and your head turns to one side.

After the seizure you feel tired but can remember exactly what happened during the seizure.

Because you do not know when a seizure might take place, you regularly feel worried and frustrated.

You regularly feel socially isolated and distant from your friends and cannot take part in a lot of your normal activities.

You also regularly have problems concentrating, and do not feel fully in control of your life.

**Health State B7**

You have at least one seizure a week, each of which lasts three minutes.

When a seizure happens, you first experience a feeling of déjà vu and a sudden change in your mood.

Following this, your muscles randomly contract and become stiff, you start to chew, mumble and smack your lips and you are unaware of the surrounding environment.

After the seizure, you feel tired and confused, and cannot remember exactly what happened during the seizure.

Because you do not know when a seizure might take place, you constantly feel depressed and angry, physically weak and have a lack of energy.

You also constantly feel socially isolated, find it extremely difficult to maintain social relationships, and cannot take part in the majority of your normal activities.

You also have serious problems concentrating and do not feel any control over your life.

**Health State B6**

You have at least one seizure a week, each of which lasts one minute.

When a seizure happens, a twitching begins in one hand, and spreads to other parts of your body.

During the seizure, you stop speaking and your head turns to one side.

After the seizure you feel tired but can remember exactly what happened during the seizure.

Because you do not know when a seizure might take place, you constantly feel depressed and angry, physically weak and have a lack of energy.

You also constantly feel socially isolated, find it extremely difficult to maintain social relationships, and cannot take part in the majority of your normal activities.

You also have serious problems concentrating and do not feel any control over your life.
