# Supplementary material for: A standard gamble study to determine health state utilities associated with seizures in glioma in the UK
Source: Health Qual Life Outcomes. 2025 Mar 11;23:22. doi: 10.1186/s12955-025-02348-0 (PMC11900579; doi:10.1186/s12955-025-02348-0)
Supplement: Supplementary file 3 — Supplementary Material 3 [file 12955_2025_2348_MOESM3_ESM.docx]

**Appendix 3 – Health States Valued Using Chained Standard Gamble**

**Health State C1 (Anchor State)**

You have severe problems walking about

You are unable to wash or dress yourself

You have severe problems doing your usual activities

You have moderate pain or discomfort

You are moderately anxious or depressed

**Health State C4**

At some point today, you suddenly suffer a seizure which lasts five minutes.

When the seizure happens, you first experience a feeling of déjà vu and a sudden change in your mood.

Following this, your muscles become tense, you lose consciousness and fall to the floor, causing bruising. Your limbs then bend and jerk quickly and repeatedly and you bite your tongue. During the seizure, you wet yourself.

After the seizure, you feel drowsy and confused, have a headache and are not able to remember what happened during the seizure.

**Health State C3**

At some point today you suddenly suffer a seizure which lasts three minutes.

When the seizure happens, you first experience a feeling of déjà vu and a sudden change in your mood.

Following this, you start to chew, mumble and smack your lips together, and you are unaware of the surrounding environment.

After the seizure, you feel tired and confused, and cannot remember exactly what happened during the seizure.

**Health State C2**

At some point today you suddenly suffer a seizure which lasts one minute.

A twitching begins in one hand, and then spreads up the same side of your body.

During the seizure, you stop speaking and your head turns to one side.

After the seizure you feel tired but can remember exactly what happened during the seizure.
